# IgG Antibodies to Pneumococcal Serotypes 1 and 5 in Relation to PCV13 Vaccination Status in Children Aged Under 5 Years in Lao PDR: A Cross-Sectional Survey

**DOI:** 10.3390/vaccines13080873

**Published:** 2025-08-18

**Authors:** Zheng Quan Toh, Ke Xin Tang, Keoudomphone Vilivong, Jana Lai, Toukta Bounkhoun, Valin Chanthaluanglath, Anisone Chanthongthip, Anne Balloch, Paul N. Newton, Audrey Dubot-Pérès, David A. B. Dance, Paul V. Licciardi, Fiona M. Russell

**Affiliations:** 1 Infection, Immunity and Global Health, Murdoch Children’s Research Institute, Royal Children’s Hospital, Parkville, VIC 3052, Australia; 2 Department of Pediatrics, The University of Melbourne, Parkville, VIC 3052, Australia; 3 Microbiology Laboratory, Lao-Oxford Mahosot Hospital Wellcome Trust Research Unit (LOMWRU), Mahosot Hospital, Vientiane 01005, Laos; 4 Centre for Tropical Medicine and Global Health, University of Oxford, Oxford OX3 7LG, UK; 5 Unite des Virus Emergents (UVE: Aix-Marseille Univ, Universita di Corsica, IRD 190, Inserm 1207), 13005 Marseille, France; 6 Faculty of Infectious and Tropical Diseases, London School of Hygiene and Tropical Medicine, London WC1E 7HT, UK

**Keywords:** pneumococcus, serosurvey, IgG, correlate of protection, pneumococcal conjugate vaccine, Lao PDR

## Abstract

Background/Objectives: Pneumococcal serotypes 1 and 5 are associated with invasive pneumococcal disease (IPD). However, data on the circulation of these serotypes in Asia following the introduction of the pneumococcal conjugate vaccine (PCV) is limited. The Lao People’s Democratic Republic (Lao PDR) introduced PCV13 into its national immunisation programme in 2013. We undertook a serosurvey to assess the IgG responses to serotypes 1 and 5 from a convenience sample of children aged under 5 years in Vientiane, Lao PDR. Methods: This cross-sectional analysis used a convenience sample of the close contacts of children under five years old who had been hospitalised with acute respiratory infections between 2013 and 2016 in Vientiane, Lao PDR. Serotype-specific IgG concentrations to serotypes 1 and 5 were measured using a modified WHO ELISA method. Results: A total of 214 participants were included, 130 of whom were unvaccinated and 84 were vaccinated with PCV13. Compared to unvaccinated participants, a higher number of PCV-vaccinated participants met the IgG threshold for IPD (≥0.35 μg/mL) [41.5% (54/130) vs. 71.4% (60/84)] for serotype 1. In contrast, for serotype 5, a similar number of participants in the PCV-vaccinated and unvaccinated group met the IgG threshold for IPD (85.7% (72/84) vs. 82.3% (107/130). Among unvaccinated children, serotype 1 IgG levels peaked at 12 and 23 months at 0.49 µg/mL (95% CIs: 0.25–0.96), while serotype 5 IgG levels were similar across age groups, ranging from 0.55 to 0.79 µg/mL. Conclusions: Our findings indicate the considerable circulation of serotypes 1 and 5 within the community in Lao PDR. Ongoing surveillance is important for informing PCV vaccination strategies.

## 1. Introduction

*Streptococcus pneumoniae*, also known as pneumococcus, is a major pathogen causing lower respiratory tract infection and death globally [1]. Approximately 502,000 deaths (95% confidence intervals: 406,000–611,000) in children younger than 5 years are estimated to be caused by pneumococcus, with the majority of deaths occurring in low- and middle-income countries (LMICs) [1]. There are more than 100 pneumococcal serotypes. The first effective infant pneumococcal vaccine contained the seven commonest serotypes causing invasive pneumococcal disease (IPD) in high-income countries. Subsequent vaccines also included serotypes 1 and 5, which were common in Africa prior to PCV introduction [2]. Current licenced PCVs contain up to 20 pneumococcal serotypes, with higher valency PCVs being developed [3]. They provide serotype-specific protection against the most common pneumococcal serotypes causing IPD [4].

Serotypes 1 and 5 were responsible for periodic outbreaks in the pre-PCV era, particularly among older children [5,6]. These serotypes are more frequently associated with pneumonia and bacteraemia compared to other serotypes (i.e., 11A, 15B, and 23B) [6,7]. Furthermore, they are rarely detected in nasopharyngeal carriage, likely due to their shorter nasopharyngeal colonisation periods compared to other serotypes [8,9,10]. Serosurveys offer an alternative method for assessing pneumococcal exposure (carriage) and may be particularly useful for detecting serotypes that have a short carriage duration. Understanding changes in pneumococcal epidemiology and disease, particularly in unvaccinated or partially vaccinated populations, is crucial for informing vaccine policy and surveillance.

Based on data from previous randomised trials of PCV7 and PCV9, an aggregated serotype-specific IgG antibody concentration of 0.35 μg/mL measured by enzyme-linked immunosorbent assay (ELISA) was established as a correlate of protection against IPD [11]. However, later studies have demonstrated that the aggregate 0.35 μg/mL threshold was an imprecise predictor of the probable efficacy of individual serotypes [12] and does not accurately reflect efficacy against pneumococcal carriage. This threshold also varies across countries with different IPD burden and income levels [12,13]. Pneumococcal carriage is a prerequisite for IPD, and it is postulated that a higher concentration of pneumococcal antibodies is needed to prevent pneumococcal carriage and transmission [13]. Specifically, the IgG thresholds against pneumococcal carriage for serotypes 1 and 5 were calculated to be 0.81 µg/mL and 0.73 µg/mL, respectively [13].

The Lao People’s Democratic Republic (Lao PDR) introduced PCV13 into its national immunisation programme in 2013. The programme adopted a 3 + 0 schedule (given at 6, 10, and 14 weeks of age), as well as catch-up vaccination for children up to 12 months. Prior to the introduction of PCV13, serotypes 1 and 5 were among the most common serotypes causing IPD in adults and children in Lao PDR [14]. Since serotypes 1 and 5 are rarely carried in the nasopharynx, serosurveys may help understand the circulation of these serotypes in the community. The aim of this study was to examine serotype-specific IgG responses to pneumococcal serotypes 1 and 5 in children aged under five years in Lao PDR as part of a secondary analysis using samples from a cross-sectional study on the seroprevalence of *Haemophilus influenzae* type b in central Lao PDR [15].

## 2. Materials and Methods

### 2.1. Study Design

This cross-sectional analysis used a convenience sample from a study published by Hefele et al. [15] on the seroprevalence of *Haemophilus influenzae* type b in central Lao PDR. The study population for this study consisted of purposively selected close contacts of children under five years old who had been hospitalised with acute respiratory infections between 2014 and 2017 in Mahosot Hospital, Vientiane, Lao PDR. Close contact was defined as any child under the age of 5 years who came into contact with the case in the two weeks preceding hospitalisation. Written informed consent was obtained from the parents or guardians of the children. The study was approved by the Royal Children’s Hospital Human Research Ethics Committee (33177B; MCRI), Oxford Tropical Research Ethics Committee (1050–13; LOMWRU), WPRO Ethics Research Committee (2013.30.LAO.2.EPI), the Lao National Ethics Committee (2013–057), and the Australian National University Human Research Ethics Committee (2016/770; ANU).

### 2.2. Procedures

Following parental or guardian consent, study staff collected demographic information by interviewing the caregiver using structured questionnaires. PCV vaccination status was determined using the parent-held Mother and Child Health (MCH) handbooks or written records from immunisation registers. Following consent, a single whole venous blood sample was taken from each eligible child for antibody testing. Blood samples were centrifuged to extract serum, and the serum sample was stored in a −80 °C freezer prior to shipping on dry ice to Murdoch Children’s Research Institute for pneumococcal serotype-specific IgG testing.

### 2.3. Pneumococcal Serotype-Specific IgG ELISA

Serotype-specific IgG concentrations of serotypes 1 and 5 were measured using a modified WHO ELISA method previously described [16]. Briefly, pneumococcal serotype-specific polysaccharides were diluted in Phosphate-Buffered Saline (PBS) and coated onto medium-binding plates (Greiner Bio-OneGmbH, Kremsmünster, Austria. Serum samples, the serum standard, and control samples were diluted 1:100 in an absorption buffer (PBS foetal calf serum containing 10 μg/mL of cell wall polysaccharide and 30 μg/mL of serotype 22F) and incubated on pre-coated plates. Horseradish peroxidase-conjugated sheep anti-human IgG and 3,3′,5,5′-tetramethylbenzidine substrate solution were added for the detection. Absorbance was measured at 450 nm with a 630 nm reference filter using a microplate reader (Biotek, Winooski, VT, USA). Serotype-specific IgG concentrations of serotypes 1 and 5 for each serum sample were derived from the standard values and expressed in μg/mL.

### 2.4. Statistical Analysis

Data were entered into the Research Electronic Data Capture database and cleaned prior to analysis. The baseline characteristics of the study participants were summarised. Continuous data were reported in terms of the median and interquartile range (IQR) as they were non-parametric. Categorical data were reported using frequencies and percentages. Participants were excluded from the analysis if they could not confirm their PCV vaccination status. As there was only a small number of participants vaccinated with less than three doses (n = 11), we classified participants vaccinated with at least one dose as vaccinated. The percentage of children whose IgG antibodies for pneumococcal serotypes 1 and 5 met or exceeded the putative thresholds (0.35 µg/mL for the aggregate IPD threshold and serotype-specific carriage thresholds of 0.81 µg/mL and 0.73 µg/mL for serotypes 1 and 5, respectively), as well as the IgG antibody geometric mean concentration (GMC) and 95% confidence intervals (CIs), was summarised by vaccination status, age group (12 to <24 months, 24 to <36 months, 36 to <48 months, 48 to 64 months), and time since last vaccination (1 to <3 months, 3 to <6 months, 6 to <12 months, 12 to <24 months, and ≥24 months). Comparisons of seropositivity rates between vaccinated and unvaccinated were performed using the Chi-square test, while comparisons of GMCs were performed using a Mann–Whiney U test. All analyses were conducted using Graphpad Prism 9.

## 3. Results

This study enrolled 256 participants. Thirty-eight (14.8%) participants were excluded from the analysis because of unverified vaccination status. Additionally, four participants were excluded due to missing information regarding age and sample collection date. A total of 214 participants were thus included in the analysis, 130 of whom (60.7%) were unvaccinated and 84 (39.3%) were vaccinated. The unvaccinated group was older compared to the vaccinated group (36 months (IQR: 29.7–48) vs. 23.9 months (IQR: 17.2–28.7)) (Table 1). Most participants in the vaccinated group were aged between 12 and 35 months, whereas the majority of participants in the unvaccinated group were between 24 and 60 months (Table 1). The time of enrolment was similar between the groups. The percentage of males and females was similar between the groups, and the majority of participants were of Lao ethnicity (75.4% and 89.3% in the unvaccinated and vaccinated group, respectively). Most participants in the vaccinated group had three doses of the PCV (86.9%) (Table 1).

**Figure 1:**
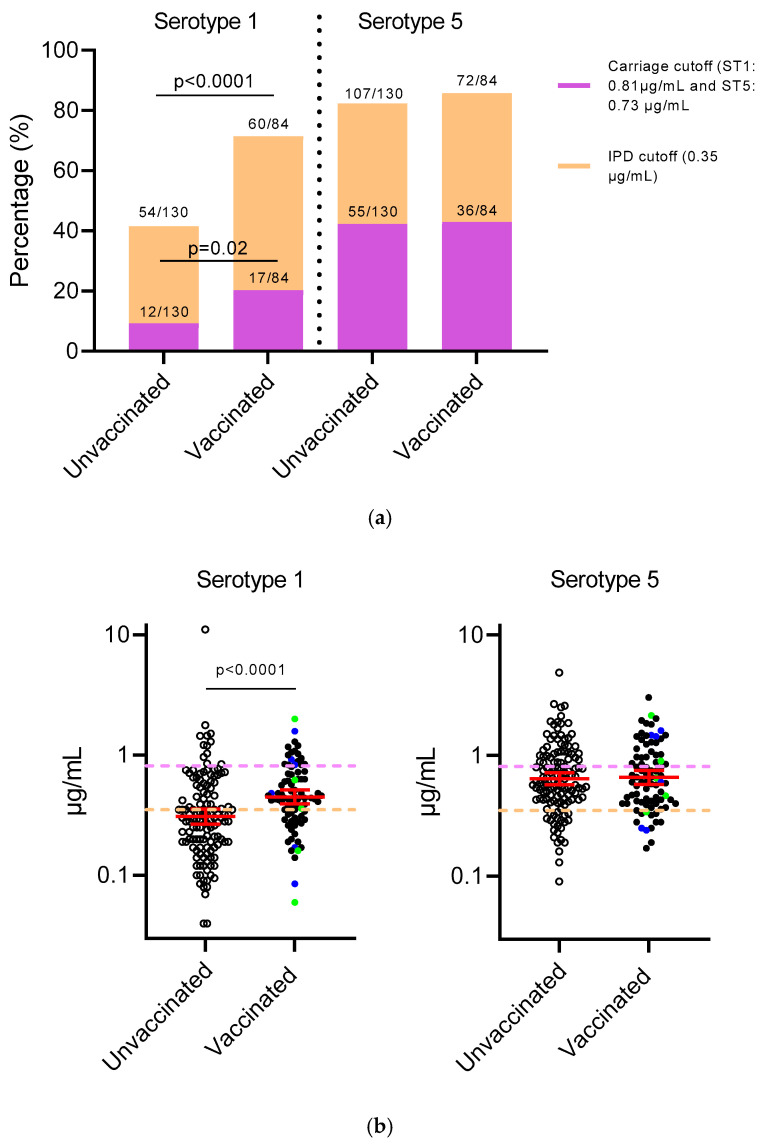
Serosurvey of pneumococcal serotypes 1 and 5 by PCV vaccination status among contacts aged 0–60 months of children hospitalised with acute respiratory infection in Vientiane, Lao PDR. (**a**) Proportion of children meeting the antibody correlates of protection based on the aggregate (0.35 µg/mL) or serotype-specific carriage (serotype 1: 0.81 µg/mL; serotype 5: 0.73 µg/mL) threshold. (**b**) Geometric mean concentrations for serotypes 1 and 5 by vaccination status. Empty and filled symbols represent children from the unvaccinated and vaccinated groups, respectively. Colour represents children vaccinated with one dose (blue) or two doses (green). Dotted lines represent the aggregate cutoff (orange) or serotype-specific cutoff (purple). Error bars (red) represent GMCs and 95% CIs.

For serotype 1, a higher percentage of children (71.4%, 60/84) in the PCV-vaccinated group met the aggregate threshold for protection against IPD (0.35 µg/mL) compared with children in the unvaccinated group (41.5%, 54/130) (*p* < 0.0001) (Figure 1a). Similarly, the percentage of children meeting the serotype 1-specific threshold for carriage (0.71 µg/mL) was higher at 20.2% (17/84) in the PCV-vaccinated group compared with 9.2% (12/130) in the unvaccinated group (*p* = 0.02) (Figure 1a). For serotype 5, similar percentages of children in the PCV-vaccinated group (85.7%, 72/84) and unvaccinated group (82.3%, 107/130) met the aggregate threshold for IPD (Figure 1a). Although the percentage of children meeting the threshold for carriage was lower than that for IPD, a similar percentage of children in the unvaccinated (42.3%, 55/130) and vaccinated (42.9%, 36/84) groups met the serotype 5-specific threshold for carriage (0.81 µg/mL) (Figure 1a). When comparing the serotype-specific IgG GMCs, children in the PCV-vaccinated group had higher serotype 1-specific IgG than children in the unvaccinated group (0.45 µg/mL vs. 0.31 µg/mL, *p* < 0.0001) (Figure 1b). In contrast, serotype 5-specific IgG GMCs were similar between PCV-vaccinated and unvaccinated groups (0.64 µg/mL vs. 0.66 µg/mL) (Figure 1b). Overall, GMCs were higher for serotype 5 than for serotype 1.

Next, we examined the percentage of children meeting the IPD and serotype-specific carriage thresholds, as well as IgG GMCs by age group and vaccination status. In the unvaccinated group, serotype 1-specific IgG GMCs and the percentage of children meeting the IPD threshold peaked between 12 and 23 months before declining with age up to 64 months (Table 2). In contrast, in the vaccinated group, these measures peak at 24–35 months before declining, although the number of participants aged 36–64 months was small (Table 2). Unlike serotype 1, serotype 5-specific IgG GMCs and the percentage meeting both IPD and serotype-specific carriage thresholds were comparable between vaccinated and unvaccinated children and remained consistent across all age groups.

**Figure 2:**
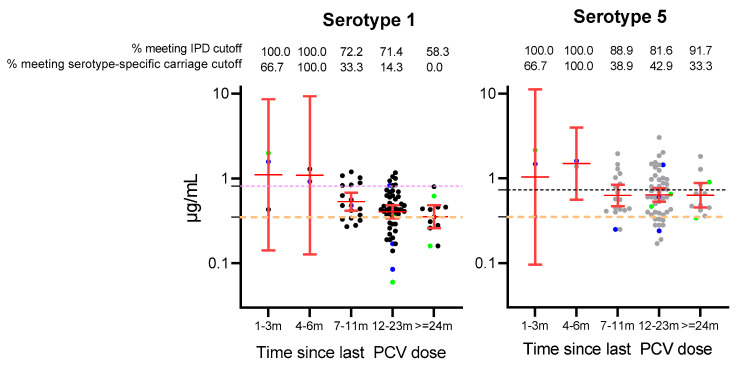
Percentage of vaccinated children meeting the correlate of protection cutoffs and geometric mean concentrations for serotypes 1 and 5 by time since last PCV dose. Black and grey represent individual participant serotype 1 and serotype 5 antibody levels, respectively. Colour represents children vaccinated with one dose (blue) or two doses (green). Dotted lines represent the IPD cutoff (orange) or serotype-specific carriage cutoff for serotype 1 (purple) and serotype 5 (black).

We also examined serotype 1 and 5 IgG responses among PCV-vaccinated children by time since their last PCV dose. Almost all individuals had antibodies above the aggregate and serotype-specific thresholds for serotype 1 and 5 up to 6 months post-vaccination, although this needs to be interpreted with caution due to the small number of participants at those timepoints (Figure 2). The GMCs for serotype 1 IgG were high at 1 and 6 months post-vaccination before waning 1.94-fold to 0.53 µg/mL between 7 and 11 months post-vaccination and continuing to decline 1.5-fold to 0.36 µg/mL by 24 months post-vaccination (almost at the aggregate IPD cutoff). By 24 months of age, only around half of the participants had serotype 1 antibodies above the aggregate threshold for IPD, while none had antibody levels above the serotype-specific threshold for carriage. In contrast, the GMCs for serotype 5 antibodies were high at 1 to 6 months post-vaccination before waning 2.4-fold to 0.63 µg/mL between 7 and 11 months post-vaccination, after which they remained stable up to 24 months post-vaccination. While the percentage of participants that met the serotype 5 aggregate threshold for IPD remained high (>81%) over two years, 33.3% to 42.9% of participants met the serotype 5-specific threshold for carriage two years post-vaccination.

## 4. Discussion

In this study, our serosurvey of children aged under 5 years in Lao PDR found more than 40% and 80% of unvaccinated children have pneumococcal serotype 1 and 5 antibodies, respectively. While unvaccinated children have lower pneumococcal serotype 1 antibody levels compared with PCV13-vaccinated children, antibody levels against pneumococcal serotype 5 were similar between the two groups. Our data suggest there is substantial ongoing circulation of pneumococcal serotype 5 and, to a lower extent, serotype 1 in Lao PDR among children under 5 years. These data have important implications for ongoing surveillance in high-burden pneumococcal countries that have recently introduced pneumococcal vaccination. They also highlight the value of serosurveys as a complementary tool to nasopharyngeal carriage studies, particularly in settings where IPD surveillance is limited.

Pneumococcal serotypes 1 and 5 are important serotypes that cause IPD globally [10], including Lao PDR [14]. Serotypes 1 and 5 typically have a high invasive disease potential, characterised by their detection in the tissues and fluids of IPD patients. Both serotypes 1 and 5 are in the current PCVs. In countries that have introduced PCV10/13 immunisation programmes, IPD cases caused by these serotypes have decreased [18,19]. In Lao PDR, data up to five years post-PCV13 introduction have demonstrated a direct impact on IPD (42% reduction in vaccine-type IPD) and pneumococcal vaccine-type carriage (23% reduction in vaccine type carriage) in young children [20]. No serotype 1- or 5-associated IPD has been reported in Lao PDR post-PCV introduction in 2013 [20]. Our data show that PCV-vaccinated children have higher serotype 1 antibodies than unvaccinated children and support the use of PCV to reduce IPD and potentially pneumococcal carriage caused by serotype 1.

While only a small and moderate percentage of children met the serotype-specific threshold for pneumococcal carriage for serotypes 1 and 5, respectively, it is noteworthy that the majority of participants in the PCV-vaccinated group had received their last PCV dose one to two years prior, during which time antibody levels might have waned. Indeed, among PCV-vaccinated children, both serotype 1 and serotype 5 antibody levels appeared to wane after six months post-vaccination. Notably, serotype 1 antibody levels continued to decline up to two years post-vaccination, whereas serotype 5 antibody levels remained relatively stable over the same period. This pattern, along with a high level of serotype 5 antibodies among unvaccinated children, as well as higher serotype 5 than serotype 1 antibody levels among vaccinated children (which is atypical following PCV13 vaccination [21,22]), may indicate the ongoing transmission of serotype 5. However, previous carriage surveys and serotyping of IPD cases in Laos PDR conducted during the same study period did not detect serotype 1 or 5 [20,23,24]. No outbreak of serotype 5 has been reported around the region, although both serotypes 1 and 5 have been found in IPD cases in nearby region such as Cambodia [25]. Both serotypes 1 and 5 have shorter nasopharyngeal colonisation periods (1–2 weeks shorter) than other serotypes (i.e., 19F), making them less likely to be detected in the nasopharynx [8,9,10]. Other reasons that might have contributed to the lack of detection of these serotypes include the recent use of antibiotics by patients [26], as well as missed cases due to the timing and frequency of sample collection. Therefore, relying on pneumococcal carriage by nasopharyngeal swab alone may underestimate the true burden of pneumococcal transmission.

While Lao PDR has made significant progress in IPD surveillance, especially in laboratory improvements, there are still key challenges in terms of under-detection, data gaps, and infrastructure limitations. As IPD surveillance is limited, alternative ways to estimate the burden of the disease are needed. The use of serosurveys might overcome some of the limitations of carriage surveys. The use of putative carriage-specific thresholds provides information on whether the vaccine or schedule is providing optimal immune protection for this important stage of pathogenesis. Lao PDR currently used a 3 + 0 PCV schedule (given at 6, 10, and 14 weeks of age). Considerations of a booster, including a two-dose primary series followed by a booster (2 + 1) schedule, may enhance the duration of protection against pneumococcal carriage, and this remains an important consideration. Longitudinal studies and ongoing surveillance are essential to assess population immunity and the impact of PCV vaccination, including potential serotype replacement.

This study has some limitations. First, the convenience sampling of the study participants and cross-sectional design, as well as the lack of information regarding family or sibling participation, may have resulted in selection bias and limit the generalisability of the findings to the broader population in Lao PDR. Second, the small sample size in the analysis by age group and the time interval since last PCV dose may have reduced our ability to draw definitive conclusions about age-specific immunity over time. Third, the thresholds for protection against IPD and carriage were based on antibody levels one-month post-vaccination and may vary across countries with different income levels, epidemiological settings, and population immunity. 

## 5. Conclusions

Our data suggest the ongoing circulation of serotypes 1 and 5 within the community. The low percentage of children meeting the serotype 1-specific threshold for carriage, particularly those unvaccinated underscores the need for PCV vaccination, ongoing surveillance and longitudinal studies to better understand transmission dynamics and protection against IPD. Serosurveys may offer an accessible alternative for assessing population-level exposure and immunity in resource-limited settings, especially for serotypes that are rarely carried in the nasopharynx.

## Figures and Tables

**Table 1:** Baseline characteristics of study cohort.

	Unvaccinated (N = 130)	Vaccinated (N = 84)
**Age, months (median IQR)**	36 (29.7–48)	23.9 (17.2–28.7)
**Age group, n (%)**		
- 12 to 23 months	13 (10.0)	43 (51.2)
- 24 to 35 months	39 (30.0)	30 (35.7)
- 36 to 47 months	42 (32.3)	10 (11.9)
- * 48 to ≥60 months	36 (27.7)	1 (1.2)
**^ Year of enrolment, n (%)**		
- 2014	1 (0.8%)	-
- 2015	52 (40.0%)	30 (35.7%)
- 2016	59 (45.4%)	45 (53.6%)
- 2017	18 (13.8%)	9 (10.7%)
**Sex, n (%)**		
- Female	62 (47.7)	38 (45.2)
- Male	68 (52.3)	46 (54.8)
**Ethnicity, n (%)**		
- Lao	98 (75.4)	75 (89.3)
- Hmong	32 (24.6)	8 (9.5)
- Khmu	(0)	1 (1.2)
**Number of vaccine doses, n (%)**		
- One	-	6 (7.1)
- Two	-	5 (6.0)
- Three	-	73 (86.9)

* Three participants aged 60 months and one aged 64 months in the unvaccinated group. ^ PCV13 first dose coverage by year: 79% (2014), 91% (2015), 93% (2016), 98% (2017). Data from WHO [17].

**Table 2:** Percentage of children meeting the IPD (≥0.35 µg/mL) and serotype-specific carriage threshold, and geometric mean concentrations for serotypes 1 and 5 by age group and vaccination status.

	Unvaccinated (n = 130)			Vaccinated (≥1 Dose) (n = 84)		
	IPD-Cutoff, n (%)	Serotype-Specific Carriage Cutoff, n (%)	GMCs (95% CIs)	IPD-Cutoff, n (%)	Serotype-Specific Carriage Cutoff, n (%)	GMCs (95% CIs)
**Serotype 1**						
- **12 to 23 m**	8/13 (61.5%)	1/13 (7.7%)	0.49 (0.25–0.96)	28/43 (65.1%)	10/43 (23.3%)	0.44 (0.35–0.54)
- **24 to 35 m**	17/39 (43.6%)	6/39 (15.4%)	0.33 (0.25–0.43)	25/30 (83.3%)	6/30 (20.0%)	0.49 (0.40–0.60)
- **36 to 47 m**	16/42 (38.1%)	4/42 (9.5%)	0.29 (0.23–0.37)	6/10 (60.0%)	0/10 (0.0%)	0.35 (0.27–0.47)
- **48 to ≥60 m**	13/36 (36.1%)	1/36 (2.8%)	0.26 (0.21–0.34)	1/1 (100.0%)	1/1 (100.0%)	0.92
**Serotype 5**						
- **12 to 23 m**	11/13 (84.6%)	6/13 (46.2%)	0.66 (0.45–0.96)	37/43 (86.0%)	20/43 (46.5%)	0.68 (0.56–0.83)
- **24 to 35 m**	28/39 (71.8%)	14/39 (35.9%)	0.55 (0.43–0.71)	25/30 (83.3%)	11/30 (36.7%)	0.60 (0.47–0.77)
- **36 to 47 m**	35/42 (83.3%)	13/42 (31.0%)	0.61 (0.50–0.74)	9/10 (90.0%)	4/10 (40.0%)	0.68 (0.46–1.00)
- **48 to ≥60 m**	33/36 (91.7%)	22/36 (61.1%)	0.79 (0.65–0.96)	1/1 (100.0%)	1/1 (100.0%)	1.61

## Data Availability

The data presented in this study are available upon request from the corresponding author.
